# Supplementary material for: Effects of education methods on self-efficacy of smoking cessation counseling among medical students
Source: PeerJ. 2021 May 7;9:e11408. doi: 10.7717/peerj.11408 (PMC8109004; doi:10.7717/peerj.11408)
Supplement: Supplemental Information 3 [file peerj-09-11408-s003.pdf]

English - Korean codebook

pack years – 평생흡연량

smoking initiation - 흡연시기

types of tobacco used - 담배종류

smoking status - 현재 흡연량

average number of cigarettes smoked per day - 1 일 평균흡연량

number of smoking days in the last month, (smoking days) average number of cigarettes smoked per day - 최근 1 개월간 흡연일수, (흡연한 날) 1 일 평균흡연량

smoking duration in the past, average number of cigarettes smoked per day in the past, quit duration -과거 흡연기간, 과거 하루 평균 흡연량 및 금연 기간이 어떻게 됩니까?

quitting experience -금연시도경험

intend to quit smoking - 금연계획

exposure to secondhand smoke at school in the past week - 최근 7 일 간접흡연경험

12 experience of learning about the harm of smoking – 12 흡연의 해로움 배운 적

13 experience of learning about smoking cessation counseling technique – 13 금연상담기법 교육 받은 적

14 smoking prevalence among adults in Korea – 14 한국성인흡연율

15 tobacco-related mortality - 15 한국에서 1 년간 흡연으로 인해 사망하는 사람 수

16 tobacco-related illnesses - 16 흡연으로 인해 발생할 수 있는 질병

17 effect of secondhand smoke on stroke - 17 간접흡연으로 인해 뇌졸중이 발생할 수 있습니까?

18 effect of brief smoking cessation interventions - 18 의료진이 제공하는 간단한 금연 상담이 금연성공에 영향을 줄 수 있습니까?

19 five major steps to intervention (the "5 As") - 19 상담기법 5 단계는

20 nicotine dependence treatment - 20 금연치료 보조제 혹은 약물은

21 length of time after quitting when the risk of cardiovascular diseases is reduced or returns to normal - 21 심혈관질환의 위험이 비흡연자와 유사한 수준으로 감소되는 금연유지기간

22 benefits of smoking cessation in reducing sudden and premature deaths - 22 금연은 연령에 상관없이 조기사망을 감소시킵니까?

23 counseling efficacy in smoking cessation - 23 금연을 위해 도움을 필요로 하는 흡연자를 능숙하게 상담할 수 있다고 생각합니까?
