# Supplementary material for: Effects of education methods on self-efficacy of smoking cessation counseling among medical students
Source: PeerJ. 2021 May 7;9:e11408. doi: 10.7717/peerj.11408 (PMC8109004; doi:10.7717/peerj.11408)
Supplement: Supplemental Information 4 [file peerj-09-11408-s004.pdf]

Questionnaire

You will soon become a doctor. Am I ready for smoking cessation treatment? We conduct a survey on smoking-related knowledge and education on smoking cessation for medical students in Busan and Gyeongnam area. We need your active interest and participation for better education and improvement of quality in health care.

1. Please select your gender
☐ male
☐ female

2. Please enter your age
(   )

3. Please select your school
☐ Kosin University College of Medicine
☐ Dong-A University College of Medicine
☐ Pusan National University School of Medicine
☐ Inje University College of Medicine

4. Please select your academic year?
☐ Year 1
☐ Year 2
☐ Year 3
☐ Year 4

5. How many cigarettes have you smoked in your lifetime?
☐ <100 cigarettes
☐ more than 100 cigarettes
☐ Never (then, question 11)

6. When did you start smoking?
☐ ~ high school
☐ pre-med year 1
☐ pre-med year 2
☐ Year 1
☐ Year 2
☐ Year 3
☐ Year 4

7. What type of cigarette do you smoke (or had smoked)? (Multiple choices are possible.)
☐ Cigarette
☐ E-cigarette
☐ Smokeless tobacco (e.g., chewing tobacco, snuff, snus and dip)
☐ Hookah
☐ Shag (Rolled tobacco)

8. How many cigarettes, on average, do you smoke per day?
① daily (then, question 8-1)
② nondaily (then, question 8-2)
③ not now (then, question 8-3)

8-1. Daily smoker: How many cigarettes, on average, do you smoke per day?
(   )

8-2. Nondaily smoker: How many days did you smoke in the last month, and how many on average did you smoke a day on the day you smoked? (e.g., 7 days, 10 cigarettes per day)
(   )

8-3. Past smoker (then question 11): How long did you smoke in the past, and how many on average did you smoke a day in the past, and what is the duration of smoking cessation? (e.g., 5 years, 20 cigarettes per day, 3 years)
(   )

9. Do you have an experience of quit attempts?
☐ Yes
☐ No

10. Do you intend to quit smoke?
☐ Yes, I’m thinking to quit within the next month.
☐ Yes, I’m thinking to quit within the next 6 months.
☐ Yes, I’m thinking to quit someday, not within the next 6 months.
☐ No, never

11. In the last 7 days, have you ever been exposed to secondhand smoke?
☐ Yes
☐ No

12. Have you ever learned about the harm of smoking at a medical school?
☐ Yes
☐ No

13. Have you ever had a formal training in smoking cessation counseling techniques at medical schools?
☐ Yes
☐ No

14. What is the smoking rate among Korean adults? (Please write the %)
(%)

15. How many people die in one year from smoking in Korea? (Please write down the number of people)
(   )

16. What are tobacco-related illnesses? (Please write up to three)
(   )

17. Can secondhand smoke cause stroke?
☐ Strongly disagree
☐ Disagree
☐ Neither agree nor disagree
☐ Agree
☐ Strongly agree

18. Can brief smoking cessation counseling provided by health care providers affect your cessation success?
☐ Strongly disagree
☐ Disagree
☐ Neither agree nor disagree
☐ Agree
☐ Strongly agree

19. Please write five major steps to intervention (the "5 As") for clinicians to provide counseling to tobacco users who want to quit.
( )

20. What supplements or drugs can be used for smoking cessation? (Please write up to three.)
( )

21. How long does it take for the risk of cardiovascular diseases to decrease to a level similar to that of a nonsmoker?
(years)

22. Is there benefits of smoking cessation in reducing sudden and premature deaths?
☐ Strongly disagree
☐ Disagree
☐ Neither agree nor disagree
☐ Agree
☐ Strongly agree

23. Do you think you can effectively counsel a smoker who needs help to quit smoking?
☐ Strongly disagree
☐ Disagree
☐ Neither agree nor disagree
☐ Agree
☐ Strongly agree
