# Supplementary material for: Effects of education methods on self-efficacy of smoking cessation counseling among medical students
Source: PeerJ. 2021 May 7;9:e11408. doi: 10.7717/peerj.11408 (PMC8109004; doi:10.7717/peerj.11408)
Supplement: Supplemental Information 5 [file peerj-09-11408-s005.pdf]

* 표시는 필수항목임

1. 성별을 선택하십시오. *
○ 남 ○ 여

2. 나이를 입력하십시오. (만 나이를 입력하십시오.) *
(   )

3. 해당 학교를 선택하십시오. *
○ 고신대학교 ○ 동아대학교 ○ 부산대학교 ○ 인제대학교

4. 해당 학년을 선택하십시오. *
○ 본과 1학년 ○ 본과 2학년 ○ 본과 3학년 ○ 본과 4학년

5. 지금까지 살아오는 동안 피운 담배의 양은 총 얼마나 됩니까?
○ 100개비 미만
○ 100개비 이상
○ 피운 적 없음 (10번으로)

6. 흡연을 시작한 시기는 언제입니까?
○ 예과 1학년 ○ 예과 2학년 ○ 본과 1학년 ○ 본과 2학년 ○ 본과 3학년 ○ 본과 4학년
○ 의대(혹은 의학전문대학원) 입학 이전

7 피우고 있는(혹은 피웠던) 담배의 종류는 무엇입니까? (복수 선택이 가능합니다.)
○ 궐련
○ 전자담배
○ 무연담배(스누스, 스너프, 씹는 담배 등)
○ 물담배
○ 말아 피우는 담배

8. 현재 담배를 얼마나 피웁니까?
① 매일 피움 (7-1번으로)
② 가끔 피움 (7-2번으로)
③ 과거에는 피웠으나 현재 피우지 않음 (7-3번으로)

8-1. 매일 피움 (8번으로)
하루 평균 흡연량이 어느 정도입니까? (개비수를 적으십시오. 예: 20)
(   )

8-2. 가끔 피움 (8번으로)
최근 1개월간 흡연한 일수는 며칠이고 (흡연한 날) 하루 평균 흡연량은 어느 정도입니까? (예: 7일, 5개비)
(   )

8-3. 과거에는 피웠으나 현재 피우지 않음 (10번으로)
과거 흡연기간, 과거 하루 평균 흡연량 및 금연 기간이 어떻게 됩니까? (예: 5년, 20개비, 3년)
(   )

9. 금연을 시도해 본 경험이 있습니까?
○ 예 ○ 아니오

10. 담배를 끊을 계획이 있습니까?
○ 1개월 안에 금연할 계획이 있다.
○ 6개월 안에 금연할 계획이 있다.
○ 6개월 이내는 아니지만 언젠가는 금연할 생각이 있다.
○ 현재로서는 전혀 금연할 생각이 없다.

11. 최근 7일 동안, 학교 주변에서 다른 사람이 피우는 담배연기를 맡은 적이 있습니까? (간접흡연에 노출된 적이 있습니까?)
○ 예 ○ 아니오

12. 의과대학(혹은 의학전문대학원)에서 흡연의 해로움에 대해 배운 적이 있습니까?
○ 예 ○ 아니오

13. 의과대학(혹은 의학전문대학원)에서 환자에게 적용할 수 있는 금연 상담 기법에 대해 정규 교육을 받은 적이 있습니까?
○ 예 ○ 아니오

14. 한국 성인의 흡연율은 어느 정도입니까? (%를 적으십시오.)
(   )

15. 한국에서 1년간 흡연으로 인해 사망하는 사람은 몇 명입니까? (명수를 적으십시오.)
(   )

16. 흡연으로 인해 발생할 수 있는 질병에 어떤 것들이 있습니까? (최대 3가지까지 적으십시오.)
(   )

17. 간접흡연으로 인해 뇌졸중이 발생할 수 있습니까?

전혀 그렇지 않다. 1 2 3 4 5 매우 그렇다.

18. 의료진이 제공하는 간단한 금연 상담이 금연성공에 영향을 줄 수 있습니까?
전혀 그렇지 않다. 1 2 3 4 5 매우 그렇다.

19. 금연 의지가 있는 흡연자에게 시도할 수 있는 상담 기법 5단계는 무엇입니까? (각 단계를 적으십시오.)
( )

20. 금연 치료에 사용할 수 있는 보조제 혹은 약물에는 무엇이 있습니까? (최대 3가지까지 적으십시오.)
( )

21. 어느 정도 금연을 유지해야 심혈관질환의 위험이 비흡연자와 유사한 수준으로 감소합니까? (몇 년인지 적으십시오.)
( )

22. 금연은 연령에 상관없이 조기사망을 감소시킵니까?
전혀 그렇지 않다. 1 2 3 4 5 매우 그렇다.

23. 금연을 위해 도움을 필요로 하는 흡연자를 능숙하게 상담할 수 있다고 생각합니까?
전혀 그렇지 않다. 1 2 3 4 5 매우 그렇다.
